# Cellular Network Fault Diagnosis Method Based on a Graph Convolutional Neural Network

**DOI:** 10.3390/s23167042

**Published:** 2023-08-09

**Authors:** Ebenezer Ackah Amuah, Mingxiao Wu, Xiaorong Zhu

**Affiliations:** Jiangsu Key Laboratory of Wireless Communications, Nanjing University of Posts and Telecommunications, Nanjing 210003, China; f2019010106@njupt.edu.cn (E.A.A.);

**Keywords:** 4G/5G network, graph convolutional neural network, heterogeneous network, fault diagnosis

## Abstract

The efficient and accurate diagnosis of faults in cellular networks is crucial for ensuring smooth and uninterrupted communication services. In this paper, we propose an improved 4G/5G network fault diagnosis with a few effective labeled samples. Our solution is a heterogeneous wireless network fault diagnosis algorithm based on Graph Convolutional Neural Network (GCN). First, the common failure types of 4G/5G networks are analyzed, and then the graph structure is constructed with the data in the network parameter, given data sets as nodes and similarities as edges. GCN is used to extract features from the graph data, complete the classification task for nodes, and finally predict the fault types of cells. A large number of experiments are carried out based on the real data set, which is achieved by driving tests. The results show that, compared with a variety of traditional algorithms, the proposed method can effectively improve the performance of network fault diagnosis with a small number of labeled samples.

## 1. Introduction

The growth of mobile data traffic and the increase in service types have led to challenges in mobile communication network capacity. Heterogeneous cellular networks have been widely explored in the literature as a means to address the bandwidth problem and improve system capacity.

However, the expansion and complexity of heterogeneous networks have made the management and maintenance of cellular networks increasingly complex. Traditional network fault detection and diagnosis methods are primarily performed manually, making it difficult to establish the mapping relationship between network symptoms and fault categories. Additionally, these methods require significant human and material resources. Therefore, there is an urgent need to establish a fast and accurate network fault diagnosis model. In response to this need, researchers have conducted in-depth research on network fault diagnosis.

In [1], the authors proposed Adaptive Root Cause Analysis (ARCA), an automatic fault detection and diagnosis solution. This method utilizes Bayesian networks combined with expert domain knowledge to perform efficient and automatic network root cause analysis based on acquired network measurement data.

The paper in [2] introduced a Bayesian network-based network fault diagnosis method for universal mobile communication systems. They constructed an automatic diagnostic model using a naive Bayesian classifier and established a relationship between network failures and root causes.

In [3], the authors analyzed the time evolution of multiple network metrics from the perspective of time dependencies between these metrics. They explored the interdependence between network metrics of the primary cell and neighboring cells in the presence of network failures. By comparing these metrics with stored historical failures, they determined the root cause of the failure.

A comprehensive detection and diagnosis framework was proposed in [4]. The detection phase relied on radio measurements and other performance indicators, comparing them with the normal behavior obtained in the profile. The diagnosis phase utilized past fault cases to identify and understand their impact on different performance metrics.

In recent years, intelligent fault diagnosis has shifted from traditional model-based methods to data-driven approaches. Gomez-Andrades et al. [5] introduced an automatic diagnosis system for LTE (Long-Term Evolution) networks based on unsupervised learning.

Their system employed Self Organizing Maps (SOMs) and the sum of squares of deviations to ensure solution quality through an iterative process. The effectiveness of this diagnosis system was verified using real and simulated LTE data.

The focus in [6] was on fault diagnosis at the air interface. The fault diagnosis model collected user measurement data related to the radio frequency (RF) interface. It then used SOMs to analyze the specific measurements of each user and determine their RF status. The results were aggregated into cell-level indicators, allowing an analysis of the air quality of the entire cell.

In [7], the authors treated the performance tuning process of cellular networks as a reinforcement learning problem and proposed a method to improve the performance of indoor and outdoor environments. To achieve effective diagnostic performance, the authors in [8] combined SoftMax neural networks and support vector machines (SVM) in supervised learning, enabling the algorithm to be implemented effectively with off-the-shelf tools.

Zhang et al. [9] proposed the use of an improved back-propagation (BP) neural network for fault diagnosis in local area networks (LAN). Experimental results confirmed the effectiveness of the improved BP neural network in LAN fault diagnosis.

In [10], a method was proposed to identify the causes of changes in performance indicators by analyzing their correlation with multiple indicators. The diagnosis involved the use of classification/regression trees to predict the membership of event counters in one or more categories of interest for performance indicators.

The authors in [11] introduced a fault-diagnosis model that combines graph neural networks and one-shot learning to address the challenge of fault classification with limited data. The method utilizes a short-time Fourier transform to convert vibration signals into two-dimensional data, enabling feature extraction and small-sample learning.

To tackle the problem of bearing fault detection, a method called Graph Neural Network-Based Bearing Fault Detection (GNNBFD) was proposed in [12]. The method constructs a graph based on sample similarity, utilizes a graph neural network (GNN) to map the features of the samples, and incorporates information from neighboring samples. The mapped samples are then fed into a base detector for fault detection, with the top n samples exhibiting the highest outlier scores being identified as faulty.

While most existing fault diagnosis solutions utilize supervised learning algorithms in machine learning, network fault diagnosis models based on supervised learning have certain shortcomings. On the one hand, during the training process of the diagnostic model, a large amount of unlabeled sample information remains underutilized and goes to waste. On the other hand, although these methods can learn the key performance indicators (KPI) of the network and the network state, they do not consider the similarity association between unlabeled samples and labeled samples, thereby limiting the performance improvement of these models.

Furthermore, obtaining labeled data in actual networks is often time-consuming, and the cost of manual category labeling is prohibitively high, resulting in a scarcity of labeled datasets for existing networks. Manual category labeling refers to the process of assigning specific fault labels to the collected data. This manual effort can involve significant human resources and expertise, which can be expensive and may not be feasible for all organizations or researchers.

As a result, this scarcity hampers the development and training of machine learning models that require labeled data to learn and make accurate predictions. Overcoming these obstacles is crucial to improving the effectiveness and efficiency of fault diagnosis systems in networks.

Although we used GCN in our paper, there are other techniques that could have been employed for the network fault diagnosis. The following are some suggestions:

1. Bayesian Networks: Bayesian networks are probabilistic graphical models that represent variables and their dependencies using directed acyclic graphs. They can be used to model network faults and their relationships, allowing for diagnosis and prediction of faults based on observed data. However, GCN was considered in this paper for the reason that it can leverage parallel processing and optimization techniques, making them scalable and efficient for large-scale network analysis. They can handle millions of nodes and edges efficiently, which is crucial for real-world network fault diagnosis. Bayesian networks, especially when dealing with complex and large networks, can be computationally expensive and may face challenges in scalability.
2. Support Vector Machines (SVM): SVMs are supervised learning models that can classify data into different categories. They can be trained on labeled network data to detect and diagnose faults based on patterns and features extracted from the network. We chose GCNs because they have the ability to learn end-to-end representations of the network data, automatically extracting relevant features directly from the graph structure. This can be advantageous for fault diagnosis as it avoids the need for manual feature engineering. SVMs, on the other hand, typically rely on handcrafted features to perform classification or anomaly detection, which may require domain expertise and time-consuming feature engineering.

As a branch of deep learning, the Graph Convolutional Neural Network (GCN) leverages graph convolutional layers with strong learning abilities to aggregate information between nodes and their neighboring nodes. This allows the network to learn new feature representations of nodes and form a neural network model by stacking graph convolutional layers, capturing the complex nonlinear relationship between key performance indicator parameters and fault types.

Moreover, when training the GCN model, both labeled and unlabeled data are used, making GCN an unsupervised learning method that reduces the reliance on labeled data.

Simultaneously, GCN utilizes the adjacency matrix of the graph to represent the similarity between sample data, enhancing training speed and improving the model’s performance [13].

Based on the above-stated challenges, we sought to come up with a solution that would perform better than the work conducted before.

In this paper, we present a network fault diagnosis method based on Graph Convolutional Networks (GCN). The contribution of our work can be summarized as follows:

- Construction of Graph Data: Our method constructs graph data by leveraging the similarity between nodes, capturing the intricate relationships within the network.
- Utilization of GCN: The constructed graph data is then fed into GCN, enabling effective network fault diagnosis by leveraging the power of graph-based learning.
- High Diagnostic Accuracy: Experimental simulations on the drive test dataset demonstrate the effectiveness of our proposed algorithm. Even with a reduced number of training samples, our method achieves high diagnostic accuracy, outperforming traditional algorithms.

## 2. System Parameters

**Figure 1:**
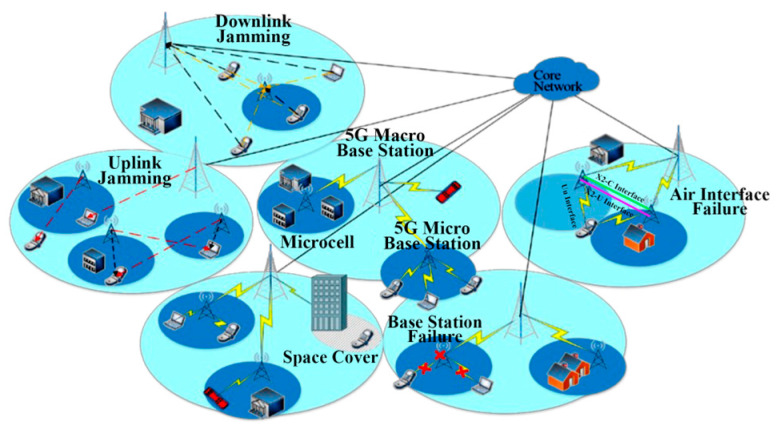
Network scene diagram.

The 5G network scenario considered in this paper is depicted in Figure 1. Within the same cell, there is an overlap between the macro base station and the micro base station. We assume that the network status can be categorized into six types, which include the normal situation, uplink interference, downlink interference, space coverage, air interface failure, and base station failure. Air interface failure results in the failure of data packet transmission, disrupting the normal communication process and causing data congestion within the network.

In most cases, base station faults are manifested by the incorrect configuration of relevant parameters. For instance, when the antenna down tilt angle parameter is excessively configured, the coverage area becomes smaller than the ideal coverage area, resulting in fewer users being served by the cell. Another example is the improper configuration of handover parameters, leading to mobility issues in a cell where successful handover cannot be achieved.

In this paper, the key performance indicators we selected are shown in Table 1, which includes 16 parameter indicators.

## 3. Feature Parameter Selection

The characteristic parameters or attributes used to train the model are referred to as “key performance index” (KPI) parameters. When dealing with a data sample containing numerous feature parameters, it becomes unfavorable for constructing the graph’s adjacency matrix, leading to an unrealistic matrix. Additionally, during the fault diagnosis stage, a large number of feature parameters can reduce the model training speed. Therefore, it is essential to obtain the optimal combination of network feature parameters based on their importance, aiming to reduce the dimensionality of input features.

To solve the optimal combination of characteristic parameters, we utilized the extreme gradient boosting algorithm (XGBoost). In each training iteration, XGBoost constructs a new model by descending the gradient of the loss function from the previously built decision tree model. This process gradually reduces the residual between the predicted value and the true value of the previous model. When constructing m decision trees, the objective function of XGBoost is represented by Equation (1):

(1) $${\mathrm{Obj}}^{\left(m\right)}=\sum_{i=1}^{N}l(y_{i},{\hat{y}}_{i}^{\left(m\right)})+\Omega (f_{m})$$

where, $l(y_{i},{\hat{y}}_{i}^{\left(m\right)})$ is the loss function, which is used to indicate the difference between the predicted network fault label ${\hat{y}}_{i}^{\left(m\right)}$ and the real network state label $y_{i}$. $\Omega (f_{m})$ is the regularization term, which is used to limit the number of sub-nodes in the decision tree and prevent overfitting in the algorithm process.

(2) $$l(y_{i},{\hat{y}}_{i}^{\left(m\right)})={\left(y_{i}-{\hat{y}}_{i}^{\left(m\right)}\right)}^{2}$$

(3) $$\Omega (f_{m})=\gamma T+\frac{1}{2}\lambda \sum_{j=1}^{T}\omega_{j}^{2}$$

where $f_{m}$ represents all *m* decision trees, *T* is the number of leaf nodes on the current decision tree, λ is the regularization parameter, *γ* is the learning rate, and $\omega_{j}$ represents the weight size of the *j^th^* leaf node on the decision tree.

${\hat{y}}_{i}^{\left(m-1\right)}$ represents the predicted value of the sample $x_{i}$ obtained by the previous model, and the second-order Taylor expansion of the objective function Obj^(*m*)^ at ${\hat{y}}_{i}^{\left(m-1\right)}$ is:

(4) $${\mathrm{Obj}}^{\left(m\right)}=\sum_{i=1}^{N}\left[g_{i}f_{m}(x_{i})+\frac{1}{2}h_{i}f_{m}^{2}(x_{i})]+\Omega (f_{m}\right)$$

where, *g_i_* and *h_i_* indicate that in the previous *m*-1 round model, the first-order derivative and the second-order derivative of the loss function of the *i^th^* sample were obtained, respectively.

(5) $$g_{i}=\partial_{{\hat{y}}^{\left(m-1\right)}}l(y_{i},{\hat{y}}^{\left(m-1\right)})$$

(6) $$h_{i}=\partial_{{\hat{y}}^{\left(m-1\right)}}^{2}l(y_{i},{\hat{y}}^{\left(m-1\right)})$$

The traversal of the samples in the objective function Obj^(*m*)^ can be converted into the traversal of the leaf nodes on the decision tree, so the objective function Obj^(*m*)^ can be rewritten as follows:

(7) $${\mathrm{Obj}}^{\left(m\right)}=\sum_{j=1}^{T}[G_{j}\omega_{j}+\frac{1}{2}(H_{j}+\lambda )\omega_{j}^{2}]+\gamma T$$

where:

(8) $$G_{j}=\sum_{i\in I_{j}}g_{i}$$

(9) $$H_{j}=\sum_{i\in I_{j}}h_{i}$$

where; *I_j_* is the set of samples on each leaf node.

Assuming that the structure of the model has been determined, the weight $\omega_{j}$ on each leaf node of the decision tree can be obtained by derivation of Equation (7) and then equated to zero, that is,

(10) $$\omega_{j}^{*}=-\frac{G_{j}}{H_{j}+\lambda }$$

Substitute the calculated weight value into the original objective function to obtain the minimum value of the objective function. Obj* is the final objective function; the smaller the value, the closer the prediction result is to the actual result, as shown in Equation (11).

(11) $${\mathrm{Obj}}^{*}=-\frac{1}{2}\sum_{j=1}^{T}\frac{G_{j}^{2}}{H_{j}+\lambda }+\gamma T$$

Based on this, we used the XGBoost model in the case of constructing multiple decision tree models. According to the classification results of the samples, the importance scores of each feature parameter can be obtained, and these feature parameters are sorted in descending order of importance scores while looking for the number of characteristic parameters in the optimal combination and finally screening the characteristic parameters from the sorted characteristic parameters. Assuming that the number of feature parameters in the original data set is *d*, after feature screening, the number of feature parameters is d_0_ (0 < *d*_0_ < *d*).

And in order not to let the higher values of these feature parameters dominate the entire training process, before using these feature parameters, it is necessary to map their values to the interval [0, 1] by a normalization method.

In this paper, the maximum value normalization is performed for each feature parameter, that is,

(12) $${\tilde{x}}_{i}=\frac{x_{i}}{\max(x_{i})},i=1,2,\ldots ,d_{0}$$

where; $x_{i}$ and ${\tilde{x}}_{i}$ represent the *i^th^* feature before and after normalization, respectively.

*Max*(*x_i_*) represents the maximum value that occurs in the *i^th^* feature attribute.

## 4. Fault Diagnosis Method Based on Graph Convolutional Neural Network

### 4.1. Generation of Graph Data

The method we proposed needs to map the dimension reduction network fault dataset {$\left(x_{1},y_{1}\right),\ldots ,\left(x_{l},y_{l}\right),\left(x_{l+1},0\right),\ldots ,\left(x_{n},0\right)\}$ to an undirected graph *G =* (*V, E*). The graph consists of two types of elements, namely node set *V* and edge set *E.*

In the data set, the class label $y_{i}\in L$, of the labeled sample, and the label set $L=\left\{1,2,\ldots ,c\right\}$ contains the class *c* network fault class label (*c* = 6).

The first *l* pieces of data $x_{i}\left(1\leq i\leq l\right)$ in the dataset are labeled data with class labels, and the remaining data $x_{u}\left(l+1\leq u\leq n\right)$ are unlabeled data, let their class labels be $y_{u}=0$. First, we construct a feature matrix $X\in R^{n\times d_{0}}$ from the dataset.

(13) $$X={\left[\begin{array}{cccc} X_{1,1} & X_{1,2} & \cdots & X_{1,d_{0}}\\ \vdots & \vdots & \ddots & \vdots \\ X_{l,1} & X_{l,2} & \cdots & X_{l,d_{0}}\\ X_{l+1,1} & X_{l+1,2} & \cdots & X_{l+1,d_{0}}\\ \vdots & \vdots & \ddots & \vdots \\ X_{n,1} & X_{n,2} & \cdots & X_{n,d_{0}} \end{array}\right]}_{n\times d_{0}}$$

The feature matrix *X* is formed by stacking and combining the feature parameter vectors of each sample node. Each row vector $X_{i}\in R^{d_{0}}$ in the matrix represents the feature parameter vector of a piece of data in the data set after removing the category label.

*n* is the number of sample nodes (the total number of samples in the network fault parameter dataset).

In order to facilitate the calculation of the cross-entropy loss function for the backward weight update of the GCN, we need a label matrix to represent the label category of the samples in the dataset. Specifically, the class labels of the labeled data in the dataset are first encoded according to the encoding style of Table 2, the class labels of the unlabeled data are represented as zero vectors, and then the label vectors of all data are stacked into a label matrix $Y\in R^{n\times c}.$

Again, an adjacency matrix $A\in R^{n\times n}$ is needed to represent the edge relationship between nodes. First, a metric $s_{i,j}(0\leq s_{i,j}\leq 1)$ based on Euclidean distance is introduced to represent the similarity measure between any two sample nodes.

(14) $$s_{i,j}=\left\{\begin{array}{ccc} \exp(-\frac{||x_{i}-x_{j}|{|}_{2}^{2}}{2\delta }) & , & \mathrm{if}(i\neq j)\\ 0 & , & \mathrm{otherwise} \end{array}\right.$$

where δ=1 is called the Gaussian bandwidth parameter. When $x_{i}$ and $x_{j}$ are more similar to each other, $s_{i,j}$ is larger; otherwise, $s_{i,j}$ is smaller.

Then, initialize and set a threshold α, if the similarity measure $s_{i,j}$ between nodes is greater than the threshold α, then the elements in the adjacency matrix $A_{i,j}=1$; if $s_{i,j}$ is less than α, then $A_{i,j}=0$.

Finally, the adjacency matrix *A* of the required graph data is obtained, and the adjacency matrix is a symmetric matrix.

(15) $$A_{i,j}=\left\{\begin{array}{c} 1\;,\;if\;s_{i,j}\geq \alpha \\ 0\;,\;otherwise \end{array}\right.$$

As for how to reasonably select the value of α, specifically, first initialize the threshold α to 0.95 and then decrease it at intervals of 0.05 (for example, 0.95, 0.90, 0.85, 0.80…), according to the accurate diagnosis of the model rate, to evaluate the rationality of the threshold selection. The simulation experiments in Section 5 show that the diagnostic accuracy of the model is optimal when the threshold is 0.80.

### 4.2. Graph Convolutional Neural Networks

In recent years, researchers have introduced graph convolutional neural networks to extend CNN to non-Euclidean data, such as graph data. In order to obtain the implicit representation or label of nodes, the graph convolution operation will aggregate the node feature information of nodes in the graph and their neighboring nodes. In the convolution operation, the relationship between nodes is mined for feature representation to form new node features and update nodes. Finally, the new node features are used to predict the label of nodes. When solving node-level tasks, such as node classification, we only focused on how to learn better local expressions for each node. At this time, the pooling operation for graph-level tasks is not necessary, so we only focused on the construction of convolution operators on the graph [14].

Since the graph data does not have translation invariance, the spectral method in the graph convolutional neural network will first transform the graph signal into the spectral domain through the Fourier transform and then use the convolution in the spectral domain according to the convolution theorem. The inverse fourier transform is converted back to the original node domain, implementing the definition of graph convolution. The convolution of the signal vector x of the node in the graph signal and the convolution kernel $g_{\theta }$ in the spectral domain can be expressed as $Ug_{\theta }U^{T}x$, where *U* is the symmetrically normalized Laplacian matrix *L* of the graph and the eigenvector after eigen decomposition matrix, $L=ULU^{T}$.

$g_{\theta }$ can be thought of as a function of the eigenvalues *L* of the Laplacian matrix *L*, i.e., $g_{\theta }(L)$.

In order to satisfy the locality of the graph convolutional neural network and reduce the calculation amount of the feature vector decomposition during the original convolution operation, Defferrard et al. [15] pointed out that the truncated expansion of the Chebyshev polynomial to the Kth order can be used to fit the original convolution kernel, and the elements can be scaled to [−1, 1].

(16) $$g_{\theta }=\sum_{k=0}^{K-1}q_{k}T_{k}(\tilde{L})$$

where $\theta_{k}$ is a vector of Chebyshev coefficients. $\tilde{L}=2L/\lambda_{max}-I_{N}$ is the scaled eigenvalue diagonal matrix, $\lambda_{\max}$ and it’s the largest eigenvalue of matrix *L*, and $I_{N}$ is the identity matrix. Therefore, the convolution of the image signal by the convolution kernel filter can be rewritten as:

(17) $$g_{\theta }*x\approx \sum_{k=0}^{K-1}q_{k}T_{k}(\tilde{L})x$$

where $\tilde{L}=U\tilde{L}U^{T}$ represents the scaled Laplacian matrix. Equation (17) is the *K*-order polynomial of the Laplacian matrix *L*, which means that only the adjacent nodes that are at most K hops away from the current node are considered when information aggregation between nodes is performed in the convolution operation.

Kipf and Welling et al. [16] rewrote the definition of graph convolution as a linear function on the Laplacian matrix through a first-order approximation, that is, *K* = 2, and proposed the concept of GCN for the first time.

(18) $$g_{\theta }*x\approx q\left(I_{N}+D^{-\frac{1}{2}}{AD}^{-\frac{1}{2}}\right)x$$

Since the eigenvalues of $I_{N}+D^{-\frac{1}{2}}{AD}^{-\frac{1}{2}}$ are in the range [0, 2], repeated use of this expression can lead to the explosion or disappearance of gradients in deep neural networks. To address this problem, Kip F et al. further employed a normalization trick $I_{N}+D^{-\frac{1}{2}}{AD}^{-\frac{1}{2}}\;\overset{\tilde{A}=A+I_{N}}{\to }{\tilde{D}}^{-\frac{1}{2}}{\tilde{A}\tilde{D}}^{-\frac{1}{2}}\;,$ where $\tilde{D}$ is the degree matrix of matrix $\tilde{A}$.

Therefore, the convolution operation of the graph convolution layer can finally be expressed as:

(19) $$H^{\left(l+1\right)}=\sigma \left({\tilde{D}}^{-\frac{1}{2}}\tilde{A}{\tilde{D}}^{-\frac{1}{2}}H^{\left(l\right)}W^{\left(l\right)}\right)$$

where, $H^{\left(l\right)}$ is the output feature matrix of the *l^th^* layer; σ represents the activation function; $W^{\left(l\right)}$ is the trainable weight matrix of the *l^th^* layer.

### 4.3. Building a Fault Diagnosis Model Based on Graph Convolutional Neural Network

In this paper, we regard network fault diagnosis as a classification task in deep learning, and the graph convolutional neural network is used to classify the nodes of the dataset samples, and the cross-entropy loss function is used as the optimization goal of the graph convolutional neural network.

During the convolution process, GCN obtains the adjacent nodes of the current node according to the adjacency matrix and then aggregates and weighs the feature attributes of the current node itself and the feature attributes of the adjacent nodes so as to obtain a new feature expression of the node. These new features will then pass through the activation function (for example, the rectified linear unit, ReLU), aggregate the attributes of the more distant and near nodes by stacking multiple graph convolution layers to gradually obtain the higher-level features of the node, and use these high-level features to complete the node classification task.

In the training process of the model, with the help of graph data, GCN learns a new feature representation of a node and then updates the weight by backpropagating the cross-entropy loss. The neighboring nodes of the current node are likely to be unlabeled. At this time, GCN uses the feature attribute information of unlabeled samples, so the training set contains labeled and unlabeled data. From this perspective, GCN is semi-supervised learning, so it reduces the use of labeled data so that the model can also achieve good diagnostic accuracy when using small sample sizes for training.

According to the above, using the network fault data set after dimension reduction, the features of these nodes are converted into an *n* × *d*_0_-dimensional feature matrix *X*, and then an n×n-dimensional adjacency matrix *A* is constructed according to the similarity between nodes. Take *X* and *A* as inputs to GCN.

(20) input=(X,A)

As mentioned earlier, the forward excitation propagation formula defined by GCN is:

(21) $$H^{\left(l+1\right)}=\sigma \left({\tilde{D}}^{-\frac{1}{2}}\tilde{A}{\tilde{D}}^{-\frac{1}{2}}H^{\left(l\right)}W^{\left(l\right)}\right)$$

In Equation (21), σ is the activation function. $\tilde{A}=A+I_{N}$, since the adjacency matrix *A* only contains the connection information of each node and the adjacent nodes in the graph, after adding the unit matrix *I_N_*, the graph convolution operation can aggregate the feature attribute information of the node itself and the adjacent nodes.

$\tilde{D}$ is the degree matrix of matrix $\tilde{A}$, with the values on the diagonal being the degrees of each node and the rest of the elements being 0. $W^{\left(l\right)}$ is a trainable weight matrix in the *l^th^* layer, which acts as a filter parameter matrix, and the parameters in it can be updated by back-error propagation.

$H^{\left(l\right)}$ is the output feature matrix of the *l^th^* graph convolutional layer for the input layer, $H^{\left(0\right)}$ is equal to the initial node feature matrix *X*. Each graph convolution layer in GCN only aggregates the node feature attributes of nodes in the first-order neighborhood of the current central node. Therefore, if you want to aggregate the node feature information of adjacent nodes in the multi-hop neighborhood to obtain higher-level node features, you need to stack a multi-layer graph convolution layer to achieve this.

The output of the graph convolutional neural network is a node feature matrix, $Z\in Rr^{n\times c}$ where *c* is the number of predefined network fault classes, i.e., *c =* 6.

**Figure 2:**
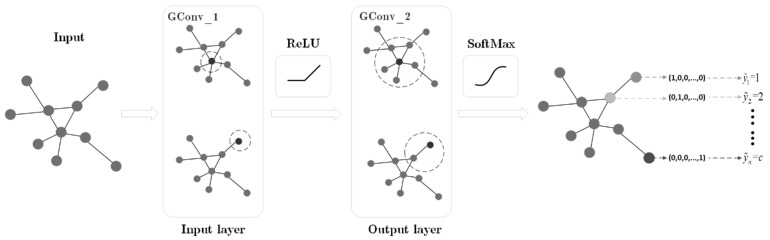
Graph Convolutional Neural Network.

To further illustrate the structure and workflow of GCN, Figure 2 shows a GCN model that contains a total of two graph convolutional layers.

As shown in Figure 2, we first calculate $\hat{A}={\tilde{D}}^{-\frac{1}{2}}\tilde{A}{\tilde{D}}^{-\frac{1}{2}}$ in Equation (21), $\hat{A}$ represents the normalized symmetric adjacency matrix, and can prevent numerical instability in the convolution operation. Since the matrix $\hat{A}$ contains the associated information of each node itself and its adjacent nodes, $\hat{A}X$ can aggregate the feature attributes of each node and the adjacent nodes. Next, a new set of node features *ÂXW*^(0)^ is obtained by multiplying with the trainable weight matrix $W^{\left(0\right)}$. Finally, an activation function is selected for the new feature matrix to obtain the output feature matrix $H^{\left(1\right)}$ of the first graph convolutional layer; that is, the new node feature representation learned by the first graph convolutional layer is given as follows:

(22) $$H^{\left(1\right)}=ReLU\;(\hat{A}XW^{\left(0\right)})$$

Stacking multiple graph convolutional layers will aggregate the feature attribute information of neighboring nodes in higher-order neighborhoods. Therefore, the output $H^{\left(l\right)}$ of the previous graph convolutional layer is used as the input of the second graph convolutional layer. After passing through the second graph convolutional layer, another set of node features $\hat{A}H^{\left(1\right)}W^{\left(1\right)}$ is learned.

However, it should be noted that since the GCN in Figure 2 uses only two graph convolutional layers, the output feature matrix of the second graph convolutional layer should have the same size as the label matrix *Y*; more specifically, this feature vector dimension of the node in the time graph changes to *c*.

Finally, the feature matrix is input into the SoftMax activation function for calculation, and the final output node feature matrix is:

(23) $$Z=SoftMax(\hat{A}H^{\left(1\right)}W^{\left(1\right)})$$

where $W^{\left(1\right)}$ is the weight matrix of the second graph convolutional layer. The SoftMax activation function needs to be applied to each row of the feature matrix $\hat{A}H^{\left(1\right)}W^{\left(1\right)}$.

For the matrix $Z=[Z_{1},\;Z_{2},\ldots ,Z_{n}]$, its form is similar to the label matrix *Y*, and each row vector $Z_{i}\left(1\leq i\leq n\right)$ in *Z* corresponds to the predicted final network fault category of the sample node $x_{i}$ in the original dataset. Specifically, for the row vector $Z_{i}=[Z_{i,1},\;Z_{i,2},\ldots ,Z_{i,c}]$, the predicted label ${\tilde{y}}_{i}={argmax}_{j}Z_{i,j}$, 1≤j≤c of the sample node $x_{i}$.

At the same time, in the GCN training process, it is necessary to calculate the cross-entropy loss function for the labeled sample nodes in the training set and use the gradient descent method to update the weights of the weight matrices in the convolutional layers of each graph through error back propagation.

(24) $$L=-\sum_{i=1}^{l}\sum_{j=1}^{c}Y_{i,j}\ln Z_{i,j}$$

where *l* is the number of labeled samples, *c* is the total number of previously defined fault classes, and *Y* is the label matrix of the previously defined nodes.

### 4.4. GCN Fault Diagnosis Process

In [17], the authors proposed a novel method for transformer fault diagnosis using a graph convolutional network (GCN) to enhance accuracy. The method combined the advantages of existing techniques, utilizing the adjacency matrix of GCN to represent similarity metrics and employing graph convolutional layers for strong feature extraction and classification. The results demonstrated the superiority of GCN over other methods such as convolutional neural networks, support vector machines, and Siamese networks in terms of diagnostic accuracy for different input features and data volumes.

The training process of GCN mainly includes two processes: forward excitation propagation and backward weight update. For the forward excitation propagation process, the input features of the sample data are processed by the multi-layer graph convolutional layer and transferred to the final SoftMax layer to obtain the output matrix *Z*, and the sample labels to be predicted are represented by the corresponding row vectors in the matrix *Z*. For the backward weight update process, the diagnostic results and the actual results are used to calculate the cross-entropy loss function, transfer the error from the output layer to the hidden layer, and then use the gradient descent method to update the weights of the weight matrices of each layer. When the model is trained, the remaining test set is used to evaluate the performance of the GCN. The process of network fault diagnosis algorithm based on GCN is shown by Algorithm 1.
**Algorithm 1** Network Fault Diagnosis Algorithm Based on Graph Convolutional Neural NetworkA. Graph data construction Input: network failure dataset after dimensionality reduction, Gaussian bandwidth parameter δ, threshold α; Output: feature matrix *X*, label matrix *Y* and adjacency matrix *A*. 1. The network failure dataset after dimensionality reduction {(*x*_1_, *y*_1_),…,(*x_l_*, *y_l_*), (*x_1_*_+1_, 0),…, (*x_n_*, 0)} The eigenvectors of each sample are superimposed onto an eigenmatrix *X*. 2. Single-hot encoding is performed on the predefined c common network faults, and the label matrix *Y* of the network fault data set is obtained according to the corresponding encoding. for *i* = 1 to *n* do  for *j* = 1 to *c* do    (1) if ***x****_i_* is labeled and *y_i_* = *j* then *Y_i_*_,*j*_ = 1 else *Y_i_*_,*j*_ = 0  end for end for 3. Construct the adjacency matrix *A* of the graph. for *i* = 1 to *n* do  for *j* = 1 to *c* and j≠i do    (1) $s_{i,j}=\exp(-\frac{||x_{i}-x_{j}|{|}_{2}^{2}}{2\delta })$, if j≠i    (2) if $s_{i,j}>\alpha$ then *A_i_*_,*j*_ = 1  else *A_i_*_,*j*_ = 0  end for end for 4. Output feature matrix *X*, label matrix *Y*, and adjacency matrix *A*. B. Network fault diagnosis based on graph convolutional neural networks Input: feature matrix *X*, label matrix *Y*, and adjacency matrix *A*; Output: set *Z* of network failures.1.Train and use the model:for *X* in *G* do                 Z←GCN(X,A)  CE loss←$L=-\sum_{i=1}^{l}\sum_{j=1}^{c}Y_{i,j}\ln Z_{i,j}$,   Error backpropagation updates the parameters of the filter matrix in each graph convolutional layer in the GCN. end for2.Fault diagnosis and classification: The fault labels of the previously unlabeled data are obtained in the output node feature matrix.

## 5. Experimental Simulation and Performance Evaluation

In practice, the rapid development of drive testing technology makes it easy to collect a large number of network sample data points, but it is time-consuming and laborious for engineers or domain experts to manually label these data points through historical experience, resulting in a low number of labeled samples in the data set. Therefore, the dataset obtained in the actual fault diagnosis often consists of a large number of unlabeled samples and a small number of labeled samples. To simulate this situation, in the following comparative simulation experiments, the training set contains only a few labeled samples.

### 5.1. Dataset Description

In this section, we used the actual dataset collected in the Nanjing Road Test in July 2019. The total number of raw data samples is 5987. In order to keep the balance of data samples for each fault type, we deleted some data samples from the same fault type.

The filtering process was performed according to the proportion of the number of samples in each category to the total number of samples in the dataset. Finally, the normal situation, uplink interference, and downlink interference are reserved, as are space coverage, air interface failures, and base station failures, which are six state types with a large proportion of data samples, including a total of 3258 samples. The specific distribution of the samples is shown in Table 3.

### 5.2. Feature Filtering

**Figure 3:**
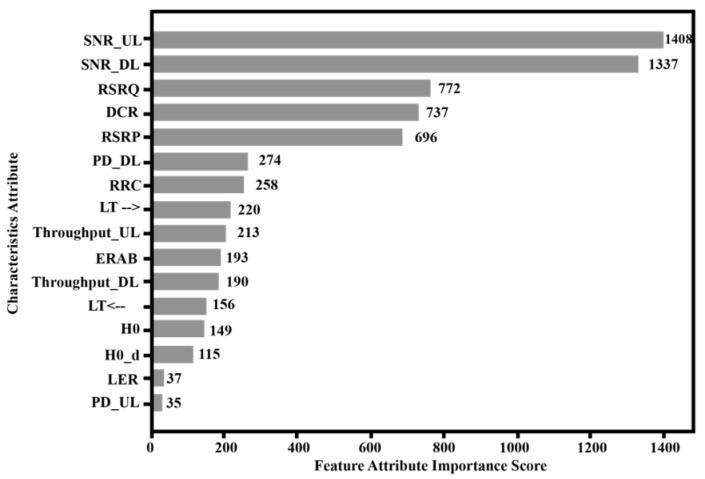
Feature Attribute Importance Score.

In order to reduce the redundant features in the sample data, make the constructed adjacency matrix more reasonable, and improve the training speed of the next GCN model. This section uses the feature screening function of the XGBoost algorithm to preprocess the original data. First, use the previous algorithm formula to give each feature attribute in the original dataset an importance score, and sort all the feature attributes according to the importance score from high to low. The simulation results are shown in Figure 3.

**Figure 4:**
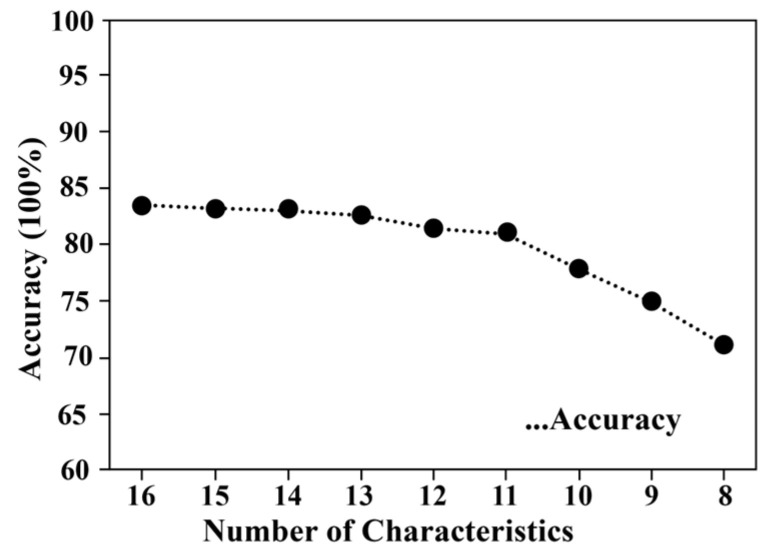
The influence of the number of features on the accuracy of XGBoost.

According to the sorted feature attributes, on the basis of ensuring that the accuracy of the model does not drop significantly, the first several feature attributes with the highest importance scores are selected as the feature attributes of the sample data after dimensionality reduction. The accuracy of the XGBoost model under different numbers of feature attribute combinations is shown in Figure 4. It can be seen that if all the features of the data are retained, the model accuracy rate is the highest; as the selected feature attributes gradually decrease, the model accuracy rate will decrease slightly; when the number of features is reduced to 11 or less, the model accuracy rate will have a sharp drop. Therefore, considering the diagnostic accuracy and the complexity of training, the top 11 features in Figure 3 are selected as the data features after dimensionality reduction.

### 5.3. GCN Parameter Settings

In order to improve the accuracy of network fault diagnosis, it is first necessary to determine the optimal structure and parameters of GCN, which mainly include the number of graph convolutional layers, the size of the filter matrix in each layer, and the threshold α when constructing the adjacency matrix A.

First, the neural network structure of GCN needs to be determined, that is, the number of layers of the graph convolution layer. Although the feature representation of the higher level of the node can be obtained by setting the multi-layer graph convolutional layer, this does not mean that the deeper the depth of the GCN, the better the effect, and a GCN model that is too deep will cause an over-smooth problem. The authors in [18] carried out explanations and experiments in this regard. They provided a deeper understanding of the graph convolutional network (GCN) model and addressed its fundamental limits. The study revealed that the graph convolution in GCNs is a form of Laplacian smoothing, which explains their effectiveness but also raises concerns about over-smoothing with multiple layers. To overcome these limits, the paper proposed co-training and self-training approaches that enhance GCNs’ performance in learning with limited labeled data and eliminate the need for additional validation labels.

Based on the above, as the number of GCN layers increases, the current node also aggregates the neighboring nodes in farther neighborhoods when performing aggregation operations, and these neighboring nodes are very useful.

It may be the adjacent nodes of other nodes (in the most extreme case, each node is aggregated on the whole graph when aggregating features), resulting in all sample data in the dataset being in the process of forward excitation propagation, the end of the model. The output results tend to be consistent, reducing the classification accuracy.

**Figure 5:**
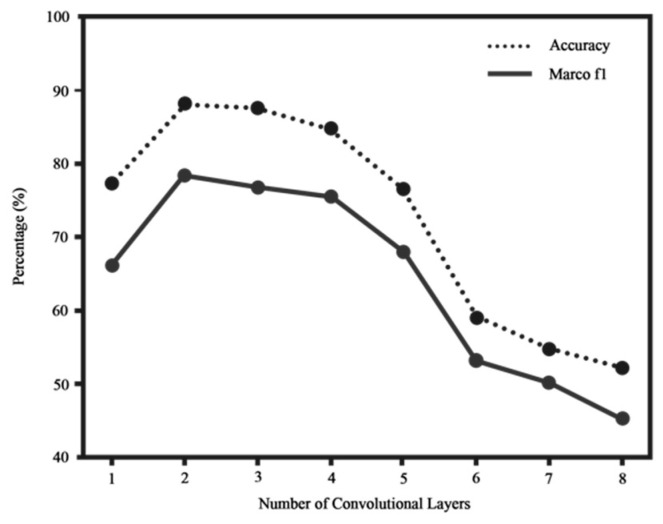
Influence of model depth on diagnostic accuracy.

This section uses two metrics, Accuracy and Macro F1, to measure how good the model is. The accuracy rate can intuitively reflect the accuracy of the model in predicting network failures, and Macro F1 takes into account the precision rate and recall rate of the classification model so that the evaluation of the model’s performance is not too one-sided. By using the dataset provided in this paper, the diagnostic accuracy and Macro F1 of the GCN model as a function of the number of graph convolutional layers are shown in Figure 5. When only one layer of graph convolutional layers is used, the model cannot well obtain the nonlinear mapping relationship between the feature attributes of the sample and its corresponding fault category; when two or three layers of graph convolutional layers are used, the diagnostic accuracy is the highest. As the number of graph convolutional layers increases, the diagnostic accuracy not only does not improve but also severely declines. Therefore, in the following simulation experiments, the model uses two layers of graph convolutional layers.

Since the input feature dimension of the sample data is 11 and the final output feature dimension is 6, the size of the convolution filter is chosen to be 7 and 6, respectively, namely $W^{\left(0\right)}\in R^{11\times 7}$ in the first layer of the graph convolutional layer and the second layer of the graph convolutional layer. In $W^{\left(1\right)}\in R^{7\times 6}$, the reason for this setting is that when passing through each layer of graph convolution, the sample data can also perform dimension reduction processing of graph node features while aggregating high-order adjacent node feature information. The input layer and the first graph convolutional layer are followed by a dropout layer with a probability of 0.25 to alleviate the overfitting problem of the model. The final GCN structure is shown in Table 4.

Finally, consider the optimal value of the threshold α when constructing the adjacency matrix A. If the value of α is set too high, the connection relationship will exist only when the similarity between nodes is very high. In this case, some connected edges between nodes that do exist may be missed, resulting in a generated adjacency matrix that cannot truly reflect each node. The similarity relationship with other nodes reduces the accuracy of the model; if the value of α is too small, the number of edges in the graph will be too large. At this time, the training speed of the model will be very slow, overfitting will occur, and the model cannot classify the samples well. The specific experimental results are shown in Table 5. In the simulation experiment, corresponding adjacency matrices are generated for different values, and then the corresponding diagnostic accuracy is obtained according to the GCN model determined above. After 10 experiments, the average value is obtained, and compared. It can be seen from Table 5 that when the value of α is 0.80, the effect of the model is the best.

### 5.4. Comparative Experimental Analysis

**Figure 6:**
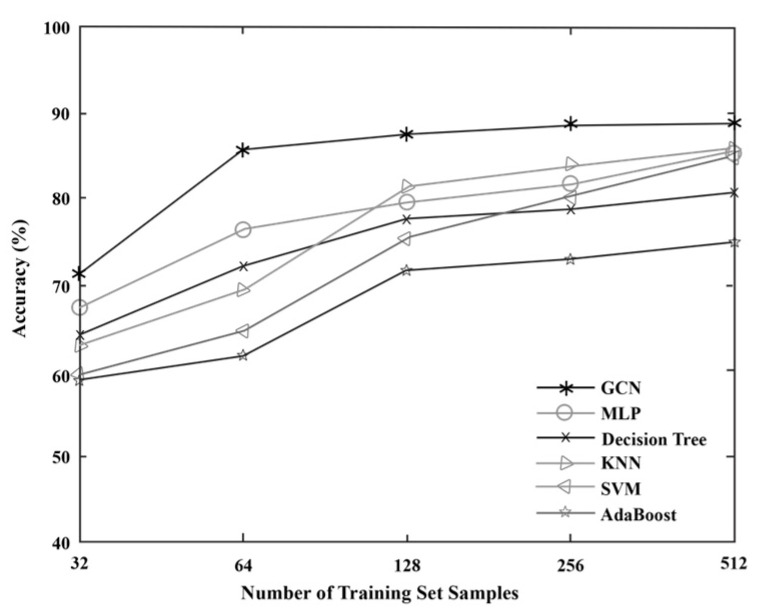
Results comparison using Accuracy in the case of small samples.

**Figure 7:**
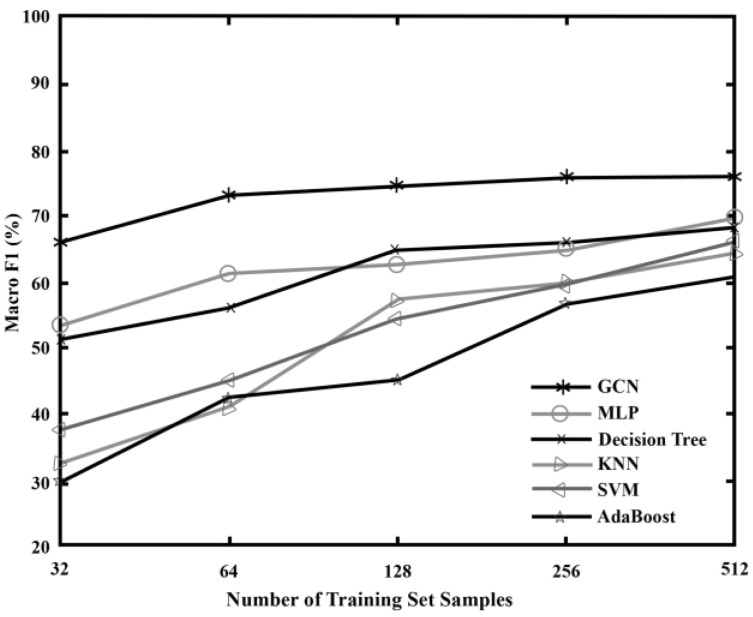
Results comparison using Macro F1 in the case of small samples.

In order to evaluate the effectiveness of the proposed algorithm model, GCN is compared with several other traditional algorithms. Since GCN is a semi-supervised learning method, both labeled and unlabeled data are used when training the model, so when discussing the training set of GCN, only the number of labeled samples in the training set is considered. A total of five sets of comparative experiments are set up in this section, and the number of labeled samples in the training set of each set of experiments is 32, 64, 128, 256, and 512, respectively. Each training set contains sample data of all network state types, and the remaining data is used as a test set to evaluate the performance of the model. Considering the actual situation, the datasets collected in the fault diagnosis process usually consist of a large number of unlabeled samples and a small number of labeled samples; therefore, experiments with more than 512 labeled samples in the training set are no longer performed in this section. Under different training sets, the diagnostic accuracy and Macro F1 of each algorithm are shown in Figure 6 and Figure 7, and the results of each group of experiments are the average of 10 repeated experiments. It can be seen that GCN has higher diagnostic performance than other algorithms under five different training sets. At the same time, by observing the data in Figure 6, it can be found that when the amount of labeled data used for training is too small, the accuracy of all algorithm models is not high due to insufficient labeled information obtained by the model. However, with the increase in training set size, the accuracy of each algorithm model on the test set is significantly improved because the information learned from the training samples is more abundant, which improves the classification ability of the model. Moreover, when the training set contains 512 samples, that is, when the training set samples account for about 16% of the data set samples, the diagnostic accuracy of GCN can reach a high level, while the rest of the algorithms are basically supervised learning methods. Therefore, neither Accuracy nor Macro F1 as compared to GCN are ideal without a large amount of labeled data for model training. This shows that GCN can also achieve good network fault diagnosis accuracy with a small number of training samples.

## 6. Conclusions

In this paper, we propose a network fault diagnosis method based on GCN. Our approach constructs graph data based on the similarity between nodes and finally inputs the graph data into GCN for network fault diagnosis. The validity of the method is verified by the experimental simulation on the drive test dataset using the model and the comparison with the traditional algorithm. Our simulation results show that the proposed algorithm can achieve high diagnostic accuracy even when using fewer samples for training.

However, when new samples need to be added to the dataset for fault diagnosis, the GCN model needs to be retrained because the size of the adjacency matrix A depends on the number of samples. In addition, the matrix operation of GCN is time-consuming, and the storage cost of the computer is large. Therefore, how to use GCN to diagnose network faults efficiently and in real time needs to be further explored.

## Figures and Tables

**Table 1:** System KPI Parameters.

KPI	Short Name
Reference Signal Receiving Power	RSRP
Reference Signal Receiving Quality	RSRQ
Packet Drop_Uplink	PD_UL
Packet Drop_Downlink	PD_DL
Signal-to-noise Ratio_Uplink	SNR_UL
Signal-to-noise Ratio_Downlink	SNR_DL
Radio Resource Control	RRC
Evolved Radio Access Bearer	E-RAB
Dropped Call Rate	DCR
Handover Success Rate	HO
Throughput_Uplink	Throughput_UL
Throughput_Downlink	Throughput_DL
Link Throughput (Out)	LT (out)
Link Throughput (in)	LT(in)
Handover Delay	HO_d
Link bit Error Rate	LER

**Table 2:** Single Hot Encoding of Network Fault Types.

Category Label	Type of Network Failure	Coding
1	Normal Conditions	1 0 0 0 0 0
2	Uplink Interference	0 1 0 0 0 0
3	Downlink Interference	0 0 1 0 0 0
4	Space Coverage	0 0 0 1 0 0
5	Air Interface Failure	0 0 0 0 1 0
6	Base Station Failure	0 0 0 0 0 1

**Table 3:** Dataset Division of Network Fault Parameters.

Serial Number	Fault Type	Quantity
1	Normal Circumstances	1420
2	Uplink Interference	175
3	Downlink Interference	175
4	Cover The Holes	496
5	Air Interface Failure	496
6	Base Station Failure	496

**Table 4:** Graph Convolutional Neural Network Structure.

Layers	Layer (Type)	Output Feature Size
1	Input Layer	3258 × 11
2	Dropout Layer 1 (rate = 0.25)	3258 × 11
3	Graph Convolutional Layer 1	3258 × 7
4	Dropout Layer 2 (rate = 0.25)	3258 × 7
5	Graph Convolutional Layer 2	3258 × 6
6	SoftMax Layer	3258 × 6

**Table 5:** The effect of α on the accuracy of GCN.

α Value	Number of Connected Edges in the Graph	Accuracy	Macro F1
0.95	4495	73.09%	66.12%
0.90	64,203	84.46%	68.32%
0.85	223,448	87.84%	76.77%
0.80	403,692	88.06%	78.80%
0.75	556,621	86.26%	75.49%
0.70	793,262	78.72%	68.09%

## Data Availability

Not applicable.
